# Nephroprotective Effects of Two Ganoderma Species Methanolic Extracts in an In Vitro Model of Cisplatin Induced Tubulotoxicity

**DOI:** 10.3390/jof8101002

**Published:** 2022-09-24

**Authors:** Sébastien Sinaeve, Cécile Husson, Marie-Hélène Antoine, Stéphane Welti, Caroline Stévigny, Joëlle Nortier

**Affiliations:** 1 RD3-Pharmacognosy, Bioanalysis and Drug Discovery Unit, Faculty of Pharmacy, Université Libre de Bruxelles, 1050 Brussels, Belgium; 2 Laboratory of Experimental Nephrology, Faculty of Medicine, Université Libre de Bruxelles, 1050 Brussels, Belgium; 3 Laboratoire de Génie Civil et Géo-Environnement- EA 4515, Université Lille, 59000 Lille, France

**Keywords:** ganoderma, cisplatin, nephrotoxicants, nephroprotection, apoptosis, anti-inflammatory, antioxidant, β-catenin, calcium, oxidative stress

## Abstract

Although cisplatin is used as a first-line therapy in many cancers, its nephrotoxicity remains a real problem. Acute kidney injuries induced by cisplatin can cause proximal tubular necrosis, possibly leading to interstitial fibrosis, chronic dysfunction, and finally to a cessation of chemotherapy. There are only a few nephroprotective actions that can help reduce cisplatin nephrotoxicity. This study aims to identify new prophylactic properties with respect to medicinal mushrooms. Among five *Ganoderma* species, the methanolic extracts of *Ganoderma tuberculosum* Murill., *Ganoderma parvigibbosum* Welti & Courtec. (10 µg/mL), and their association (5 + 5 µg/mL) were selected to study respective in vitro effects on human proximal tubular cells (HK-2) intoxicated by cisplatin. Measurements were performed after a pretreatment of 1 h with the extracts before adding cisplatin (20 µM). A viability assay, antioxidant activity, intracytoplasmic β-catenin, calcium, caspase-3, p53, cytochrome C, IL-6, NFκB, membranous KIM-1, and ROS overproduction were studied. Tests showed that both methanolic extracts and their association prevented a loss of viability, apoptosis, and its signaling pathway. *G. parvigibbosum* and the association prevented an increase in intracytoplasmic β-catenin. *G. parvigibbosum* prevented ROS overproduction and exhibited scavenger activity. None of the extracts could interfere with pro-inflammatory markers or calcium homeostasis. Our in vitro data demonstrate that these mushroom extracts have interesting nephroprotective properties. Finally, the chemical content was investigated through a phytochemical screening, and the determination of the total phenolic and triterpenoid content. Further studies about the chemical composition need to be conducted.

## 1. Introduction

Cisplatin (cis-diamminedichloroplatinum or CisPt) is commonly used as a chemotherapeutic drug to treat numerous cancers, such as lung, testicular, or ovarian cancer [1]. Studies on CisPt have shown that its primary target is the DNA by being an alkylating agent, although the entire mechanism is not completely understood. The CisPt’s uptake leads to DNA damage, affecting RNA transcription, the cell cycle, and therefore inducing the process of apoptosis [2,3]. CisPt has not only demonstrated good efficacy but also significant toxicity, including nephrotoxicity, ototoxicity, neurotoxicity, and hepatotoxicity [4]. Among these, nephrotoxicity has been widely studied as it can lead to a discontinuation of the treatment.

This toxicity prevalence remains high, occurring in about one-third of treated patients [4]. The clinical manifestations observed are a reduced glomerular filtration rate, higher serum creatinine level, and hypomagnesemia associated with hypokalemia. CisPt accumulates mainly in the proximal tubular cells where organic cation transporters (OCTs) are expressed [5]. CisPt is mainly transported into kidney cells by OCT-2 and to a lower level by the copper transporter 1 (Ctr1), which are mostly expressed in the basolateral membrane of proximal tubules [6]. Due to this uptake, CisPt accumulates in kidney cells and can interact with various reactive groups and induces DNA damages, activating the pro-apoptotic and pro-inflammatory signaling pathways [4,5,6].

In order to counteract this nephrotoxicity and allow a full and adequate treatment in the patient, there are only some recommendations for the clinician, such as increasing the patient’s hydration, regularly checking renal functional parameters, and using lower CisPt doses [7,8]. CisPt analogs have also been synthesized, such as carboplatin and oxaliplatin, to reduce this toxicity and can be used as a combination therapy with CisPt [6,7].

In addition, there is currently a lack in new potential adjuvant treatments for preventing the tubulotoxicity induced by CisPt. Natural products have been studied to supplement this paucity and can potentially increase the patient’s treatment adherence. Medicinal mushrooms are, therefore, an interesting path to study and to obtain new potential adjuvant drugs [6,7,8]. Indeed, various species have been studied as potential nephroprotective agents in the CisPt-induced model either in vitro [9,10,11] or in vivo [12,13].

Genus *Ganoderma* P. Karst has been widely studied for its potential medicinal properties. It is estimated to comprise nearly 300 species from which 20 are intensively studied for their biological activities [14]. The Ling Zhi, a member of the genus *Ganoderma*, is well-known in Traditional Chinese Medicine and is represented in the *Chinese Pharmacopoeia*. It consists of the dried sporophores of two species: *G. lucidum* and *G. sinense* [15]. *G. lucidum* mainly and other species, such as *G. applanatum* or *G. resinaceum*, have been studied for various activities such as for their anticancer, antidiabetic, and antiviral properties [16,17,18,19,20,21]. Following the increase in interest with respect to *G. lucidum*, it was added to the *European Pharmacopoeia* (Version 10.8). *Ganoderma*’s have been studied for their rich chemical content, including mainly polysaccharides and terpenes [14]. Among these, *G. lucidum* has been already studied as a nephroprotective agent in CisPt-induced tubulotoxicity [13].

In this study, methanolic extracts of five *Ganoderma* species have been screened in vitro on Human Kidney (HK-2) cells in order to test their possibly protective effects against CisPt tubulotoxicity. It was interesting to identify if other species from the same genus could also have a nephroprotective effect. The species studied were the following: *Ganoderma tuberculosum* Murill., *G. applanatum* Pat., *G. parvigibbosum* Welti & Courtec., G. *martinicense* Welti & Courtec., and *G. resinaceum* Boud. Among them, two belong to the list of the twenty most-studied [14], while the others are less known for their potential bioactivity. On top of that, to the best of our knowledge, *G. parvigibbosum* has not been studied yet and is, therefore, interesting for identifying new medicinal species. Targeted tests on the most promising species have then been performed to characterize the mechanisms of observed protection by evaluating, in particular, antioxidant, anti-inflammatory, and antiapoptotic potentials.

## 2. Materials and Methods

### 2.1. Reagents and Culture Media

Ascorbic acid, anhydrous sodium carbonate, sodium citrate, copper (II) sulfate pentahydrate, iron (III) chloride, magnesium shreds, bismuth nitrate, mercuric chloride, iodine, potassium iodide, sulfuric acid, chlorohydric acid, chloroform, DMSO, Fluo-3 AM solution, Folin Ciocalteu’s reagent, glacial acetic acid, methanol, vanillin, 2′,7′-dichlorodihydrofluorescein diacetate (H_2_DCF-DA), and 2,2′-diphenyl-1-picrylhydrazyl (DPPH) were purchased from Sigma-Aldrich, Overijse, Belgium.

Quercetin dihydrate was obtained from Riedel de Haën, Selze, Germany.

Accutase, Dulbecco’s Modified Eagle Medium (DMEM) low glucose, Dulbecco’s PBS, Fetal bovine serum (FBS), L-glutamine, and Penicillin/Streptomycin solution were acquired from Capricorn Scientific, Ebsdorfergrund, Germany.

Anti-human β-catenin-phycoerythrin monoclonal antibody was purchased from R&D Systems, Abingdon, UK.

Pluronic F127 was obtained from Thermo Fisher, Geel, Belgium.

FITC Annexin V Apoptosis Detection Kit I, Cytofix/Cytoperm solution, anti-human IL-6-APC, anti-human KIM-1-phycoerythrin, and anti-human NFκB-Alexa Fluor 488 monoclonal antibodies were acquired from BD Biosciences, Franklin Lakes, NJ, USA.

Cell Counting Kit-8 (CCK-8) assay was purchased from the Dojindo Laboratories, Kumamoto, Japan.

CisPt solution was obtained from TEVA Pharmaceutical, Antwerpen, Belgium.

Anti-human p53-Alexa Fluor 647, anti-human caspase-3-phycoerythrin, and anti-human cytochrome C-FITC monoclonal antibodies were acquired from Santa Cruz Biotechnology, Dallas, TX, USA.

### 2.2. Sample Preparation

Seven specimens belonging to five *Ganoderma* species were collected. The species are namely two *G. tuberculosum* (G. tub.), two *G. applanatum* (G. app.), one *G. parvigibbosum* (G. par.), one *G. martinicense* (G. mar.), and finally one *G. resinaceum* (G. res.). They were collected as follows: G. tub. in Martinique in 2006 and 2008; G. app. in Martinique in 2006 and Belgium in 2018; G. par. in Martinique in 2007; G. mar. in Martinique in 2008; and G. res. in Belgium in 2009. They were directly dried after collection and then stored and identified at the Belgian Coordinated Collection of Microorganisms/Mycotheque of the Université Catholique de Louvain (BCCM/MUCL), Belgium. Prior to tests, they were ground into a powder using a ZM 100 grinder, Retsch, Germany. An extraction of 5 g of the powdered mushrooms with 200 mL of methanol was performed under agitation thrice for 24 h. Extracts were then dried using a rotary vacuum evaporator (Büchi, The Netherlands). Stock solutions were prepared at 100 mg/mL in DMSO and stored at −20 °C. Sample solutions were obtained by dilution in Serum Free DMEM low glucose at 1 mg/mL and filtered through a 0.2 µm cellulose acetate membrane (VWR, Radnor, PA, USA) before being diluted to reach a working concentration.

### 2.3. Cell Culture and Treatments

Human proximal tubular epithelial (HK-2) cells were obtained from the American Type Culture Collection (ATCC, Virginia, USA). They were grown in low glucose DMEM containing 10% FBS, 1% L-glutamine, and 1% penicillin–streptomycin. Cells were subcultured and harvested for experiments every week. Cells were used between passages 8 and 15 for experimental purposes, harvested using accutase, and seeded in 12-well plates (1 or 2 × 10^5^ cells) or 96-well plates (1 × 10^4^ cells). Cells were then incubated for 24 h in complete medium, rinsed twice with DMEM, and treated with test substances for 24 to 48 h in FBS-free medium.

Four groups were compared in each experiment: control (untreated), negative control (10 µg/mL mushroom extract treated), positive control (20 µM of CisPt), and tested group (pre-treatment with the mushroom extract at 10 µg/mL 1 h prior to the addition of CisPt 20 µM). In the case of associations, methanolic extracts were added each at 5 µg/mL. CisPt’s concentration was chosen to reach about 85% of cell survival.

### 2.4. Cell Viability Assay (CCK-8)

The cell’s viability was determined with an extract of the 7 specimens individually as well as with the association of G. tub. and G. par. using Cell Counting Kit-8 (CCK-8). CCK-8 is a tetrazolium salt that will be reduced by metabolically active cells in a formazan, allowing an estimation of cell viability [22]. The tests took place in a 96-well for 24 h and after treatment, 10 μL of CCK-8 solution was added to each well, and the 96-well plate was continuously incubated at 37 °C for 2 h. The OD value for each well was read at a wavelength of 450 nm to determine the cell’s viability with a Labsystems iEMS reader/dispenser MF (Labsystems, Vantaa, Finland). The assay was repeated six times.

### 2.5. Scavenger Activity

The scavenger activity was assessed by the measurement of the scavenging ability of extracts towards the stable free radical 2,2′-diphenyl-1-picrylhydrazyl (DPPH). Serial dilutions (2-fold) of extracts (1 mg/mL) were mixed with 225 μL of methanolic 0.04% DPPH in 96-wells plates and left for 30 min in the dark; absorbances were measured with a Labsystems iEMS reader/dispenser MF (Labsystems, Finland) at 540 nm and 620 nm, using methanol as a blank and a 0.04% methanolic DPPH solution as the control. The assay was repeated three times, and for each, two serial dilutions of standards were added, namely quercetin dihydrate and ascorbic acid. The assay was repeated three times. The scavenger activity was calculated as described in Equation (1), the IC_50_ was calculated, and the residual DPPH free radical at working concentrations was graphically determined.

% Scavenger activity = [(OD_540_ − OD_620_)_tested_/(OD_540_ − OD_620_)_control_] × 100 (1)

### 2.6. Flux Cytometry Analyses

Cells were seeded in a 12-well plate (1 or 2 × 10^5^ cells). The analyses were performed on a BD FACSCanto II flow cytometer (BD Pharmingen, San Diego, CA); a minimum of 10^4^ cells was recorded. The mean fluorescence intensities were estimated, compared to controls, and expressed as proportions. All antibodies were used in accordance to the working concentration specified by the manufacturer. The assays were repeated three times.

#### 2.6.1. Apoptosis Detection: Annexin V/PI Assay and Estimation of the p53, Caspase 3 and Cytochrome C Pro-Apoptotic Markers

Firstly, the Annexin V/PI assay was performed. Cells were harvested after 24 h and centrifuged at 1400× *g*. Cells were then incubated with a solution of binding buffer containing the Annexin V and PI for 15 min at 4 °C. Cells were washed and analyzed. The total apoptosis was estimated by the sum of the early apoptotic cells (Annexin V positive and PI negative) and the late apoptotic cells (Annexin V positive and PI positive).

Secondly, p53, caspase-3, and cytochrome C, three markers of the pro-apoptotic signaling pathway, were estimated. For this, cells were harvested after 24 h, centrifuged at 500× *g*, and fixed in Cytofix/Cytoperm solution for 20 min at 4 °C. The suspension was washed and incubated with a solution of anti-human p53, anti-human caspase-3 and anti-human cytochrome C monoclonal antibodies for 30 min in the dark at 4 °C. Cells were then washed and analyzed.

#### 2.6.2. Oxidative Stress Measurement

The reactive oxygen species (ROS) overexpression was determined using the H_2_DCF-DA reagent, a reduced form of fluorescein, which, when cleaved and oxidized, especially by ROS, is converted into a highly fluorescent form. After 24 h of treatment conditions, cells were incubated for 30 min with a 10 µM H_2_DCF-DA solution. They were then rinsed, harvested, centrifuged at 1400× *g*, and analyzed.

#### 2.6.3. Anti-Inflammatory Potential Measurements

The anti-inflammatory potential was assessed by estimating the proportion of three pro-inflammatory markers, namely NFκB, IL-6 and KIM-1. Cells were harvested after 24 h, centrifuged at 500× *g*, and fixed in Cytofix/Cytoperm solution for 20 min at 4 °C. The suspension was washed and incubated with a solution of anti-human NFκB, anti-human IL-6, and anti-human KIM-1 monoclonal antibodies for 30 min in the dark at 4 °C. Cells were then washed and analyzed.

#### 2.6.4. Intracytoplasmic Calcium Estimation

The intracytoplasmic calcium proportion was assessed using Fluo-3 AM in order to observe the late dysregulation in its homeostasis. Cells were harvested after 48 h and centrifugated at 1400× *g*; then, the cells were incubated in 200 µL of a 1 µM Fluo-3 and 1 mg/mL Pluronic mix solution at room temperature for 30 min. Cells were washed twice and then analyzed.

#### 2.6.5. Intracytoplasmic β-catenin Estimation

The intracytoplasmic β-catenin estimation was assessed in order to estimate the cell adherence and its decrease in stress conditions. Cells were harvested after 48 h, centrifuged at 500× *g* and fixed in Cytofix/Cytoperm solution for 20 min at 4 °C. The suspension was washed twice and incubated with an anti-human β-catenin-PE monoclonal antibody in the dark for 30 min. Cells were washed twice and then analyzed.

### 2.7. Chemical Content

#### 2.7.1. Phytochemical Screening

The phytochemical screening was performed in order to identify the presence or absence of 6 types of secondary metabolites. They are namely the following: simple carbohydrates, terpenes, saponins, alkaloids, flavonoids, and tannins. For these tests, 0.5 g of the mushroom was extracted in 10 mL of methanol for one hour in an ultrasonic bath. Extracts were then filtered using Büchner filtration, and volumes were then completed at 10 mL. All tests were performed by adapting methods from the literature [23,24,25].

The presence of simple reducing carbohydrates was assessed using the Benedict’s test. For this, 2 mL of the Benedict’s reagent (1 g anhydrous sodium carbonate, 1.73 g of sodium citrate, and 0.173 g of copper (II) sulfate pentahydrate in 10 mL of water) was added to 1 mL of the extract. The mixture was then heated in a boiling bath for 3 min. The color obtained determined the presence or absence of simple reducing carbohydrates (blue: none; green: traces; orange: moderate; red: large amount)

The terpenes’ presence was determined by the Salkowski’s test. To 1 mL of extract, 3 mL of chloroform were added, followed by few drops of sulfuric acid. A red–brown color appearing between the two phases confirms the presence of terpenes.

The presence of saponins was permitted by the foam test. Firstly, 1 mL of extract was evaporated. Then, the residue was then dissolved in 10 mL of water and vigorously agitated. If a minimum of 1 cm of persistent foam (>10 s) formed, the test was considered positive with respect to the presence of saponins.

The alkaloids’ presence was estimated by three reagents: Dragendorff, Mayer, and Bouchardat. The apparition of a precipitate after the addition of a few drops of reagent to 1 mL of extract allowed the confirmation of the presence of alkaloids.

The presence of flavonoids was assessed by the Shinoda test. To 1 mL of extract, 1 mL of hydrochloric acid was added in addition to 1 mL of water and a few magnesium shreds. Hydrogen gas was produced and a red coloration of the solution indicates the presence of flavonoids.

The tannins’ presence was determined by adding a few drops of 5% aqueous ferric chloride solution (*m*/*V*). The apparition of a brown–black or blue–green precipitate indicates the presence of tannins.

#### 2.7.2. Total Phenolic Content

The total phenolic content (TPC) was determined following the Folin Ciocalteu’s method. The method was adapted from Rojo-Poveda et al. [26]. Briefly, 100 µL of Folin 10% solution was added to 20 µL of standard or to a 1 mg/mL methanolic extract solution in a 96-well plate. It was then incubated at room temperature in the dark for 3 min. 75 µL of a sodium carbonate 7.5% aqueous solution was then added before incubation at room temperature in the dark for one hour. Absorbance was then measured at 740 nm using a BioTek Synergy HT spectrophotometric multi-detection microplate reader (BioTek Instruments, Milan, Italy). Gallic acid was used as a standard from 0 to 700 µM. All measurements were repeated thrice.

#### 2.7.3. Total Triterpenoid Content

The total triterpenoid content (TTC) was determined by adapting the method used by Wei et al. [27]. Briefly, 200 µL of standard or 1 mg/mL methanolic extract solution was evaporated in test tubes. 1 mL of a five percent vanillin in a glacial acetic acid solution was added prior to 1.8 mL of sulfuric acid. Tubes were then heated at 70 °C for 30 min. 7.2 mL of glacial acetic acid was added before the absorbance was measured at 573 nm using a Genesys 10 spectrophotometer (Spectronic Unicam, Gent, Belgium). Oleanolic acid was used as a standard from 0 to 1000 µM. All measurements were repeated thrice.

### 2.8. Statistical Analyses

Statistical analyses were performed on results normalized and compared to the respective controls. The data were compared by means of a one-way ANOVA using GraphPad Prism 8 software (San Diego, CA, USA), and *p* values < 0.05 were considered significant.

## 3. Results

### 3.1. Cell Viability Assay (CCK-8)

**Figure 1:**
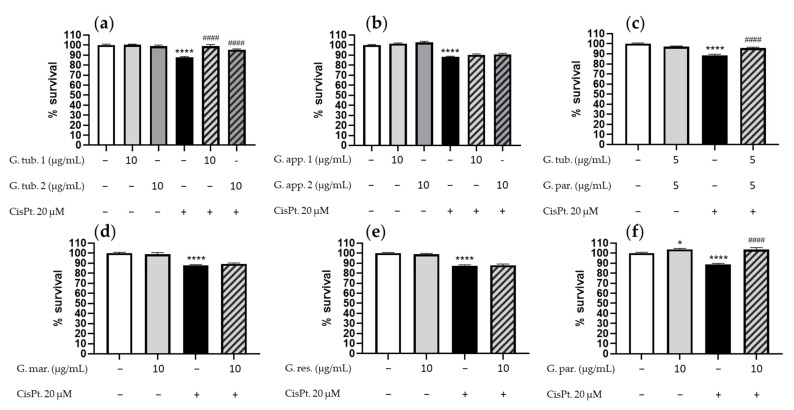
Cell survival rate determined using the CCK-8 assay after a 24 h treatment with CisPt 20 µM and/or: (**a**) one of the G. tub. extract (10 µg/mL); (**b**) one of the G. app. extract (10 µg/mL); (**c**) the G. tub. and G. par. extract (each at 5 µg/mL); (**d**) the G. par. extract (10 µg/mL); (**e**) the G. mar. extract (10 µg/mL); (**f**) the G. res. extract (10 µg/mL). Results are shown as mean ± SD of six independent experiments (**** *p* < 0.0001 and * *p* < 0.05 compared to controls; #### *p* < 0.0001 compared to CisPt. 20µM). The signs + and − indicate the presence or the absence of the corresponding treatment, respectively.

The effect on the cell viability after CisPt incubation with or without the mushroom extracts is shown in Figure 1. Results were obtained after 24 h incubation with CisPt 20 µM by the use of the CCK-8 assay. Six independent experiments were performed for each experimental condition and *Ganoderma* species.

The two G. tub. extracts did not affect the cell survival rate (Figure 1a), whereas the exposure to CisPt alone led to a significant decrease in cell survival (87 ± 6%). Both G. tub. extracts were able to significantly prevent the cell mortality (respectively 99 ± 8% and 96 ± 3%).

The two G. app., G. mar. and G. res., did not affect cell survival rate (Figure 1b,d,e), whereas the exposure to CisPt alone led to a significant decrease in cell survival (respectively 88 ± 4%; 88 ± 3% and 87 ± 5%). None of these extracts were able to significantly prevent the cell mortality (respectively 90 ± 4% and 91 ± 6 %; 89 ± 4%; 88 ± 6%).

G. par. positively affected cell survival (104 ± 4%) (Figure 1f), while the addition of CisPt led to a significant decrease (89 ± 4%). G. par. extract allowed a significant prevention of cell mortality (103 ± 5%).

These screening results led to the identification of two interesting species: *Ganoderma tuberculosum* and *Ganoderma parvigibbosum*. Following these results, G. tub. 1 and G. par were further studied and a synergetic potential was also tested between both active extracts by reaching a total concentration of 10 µg/mL with an equivalent proportion of both extracts (5 µg/mL for both). The association of G. tub. and G. par. did not affect cell survival (Figure 1c), whereas the exposure to CisPt alone led to a significant decrease in cell survival (88 ± 6%). The association allowed a significant prevention in cell mortality (96 ± 5%).

Considering these screening results, a decision was made to investigate the mechanism of action of *G. tuberculosum 1*, *G. parvigibbosum*, and their associations.

### 3.2. Apoptosis Detection: Annexin V/PI Assay and Estimation of the p53, Caspase 3, and Cytochrome C pro-Apoptotic Markers

**Figure 2:**
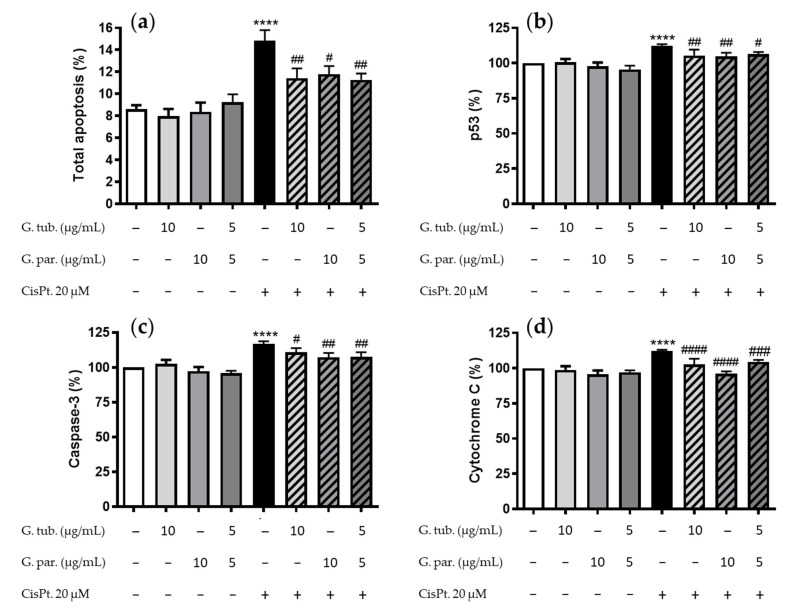
Apoptosis determinations assessed using flow cytometry of cells exposed during 24 h to CisPt 20 µM and/or the G. tub. extract (10 µg/mL), the G. par. extract (10 µg/mL), or the G. tub. and G. par. association extract (each at 5 µg/mL). (**a**) The total apoptosis process was determined following Annexin V/PI staining, and results are shown as mean ± SD of three independent experiments (**** *p* < 0.0001 compared to controls; ## *p* < 0.01 and # *p* < 0.05 compared to CisPt. 20 µM). Three markers were analyzed, and their respective normalized fluorescence compared to the controls is shown as follows: (**b**) intracytoplasmic p53 proportion, (**c**) membranous caspase-3 proportion, and (**d**) intracytoplasmic cytochrome C proportion. Results are shown as mean ± SD of three independent experiments (**** *p* < 0.0001 compared to controls; #### *p* < 0.0001, ### *p* < 0.001 ## *p* < 0.01, and # *p* < 0.05 compared to CisPt. 20 µM). The signs + and − indicate the presence or the absence of the corresponding treatment, respectively.

The total apoptosis assessment is shown in Figure 2a. The G. tub., G. par. extracts, and their association had no significant impact on the total apoptosis process compared to the controls. CisPt significantly increased apoptosis (14.9 ± 3.6%), and this increase was significantly prevented by a pre-treatment with G. tub., G. par, and their association (11.4 ± 3.8%, 11.8 ± 2.5%, and 11.3 ± 2.2%, respectively).

Among the three markers studied, namely p53, caspase-3, and cytochrome C, the mushroom extracts did not affect their intracytoplasmic proportions compared to the controls. The p53 proportion increased up to 112 ± 5% by CisPt (Figure 2b). This increase was prevented by G. tub. (105 ± 3%), G. par. (105 ± 6%), and their association (107 ± 4%). The caspase-3 proportion increased up to 117 ± 7% by CisPt (Figure 2c). This increase was prevented by G. tub. (110 ± 7%), G. par. (107 ± 8%), and their association (107 ± 8%). The cytochrome C proportion increased up to 112 ± 4 % by CisPt (Figure 2d). This increase was prevented by G. tub. (103 ± 9%), G. par. (96 ± 4%), and their association (104 ± 3%).

### 3.3. Antioxidant Potential: Scavenger Activity and ROS Determination (H_2_DCFDA Assay)

**Figure 3:**
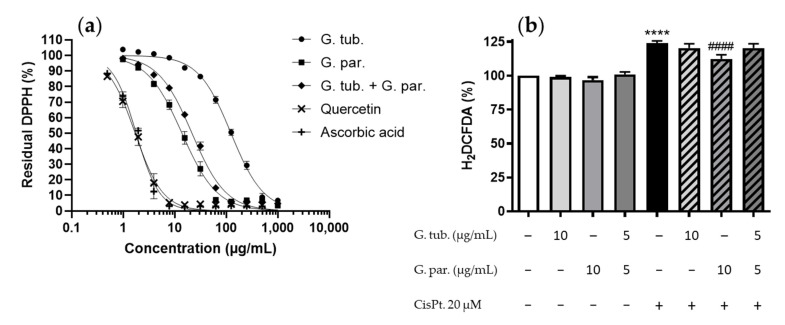
Antioxidant potential of G. tub., G. par. and their association represented by (**a**) their scavenger activity based on residual DPPH percentages on serial 2-fold dilutions (starting concentration from 1000 µg/mL; while standards were added at 500 µg/mL); (**b**) normalized fluorescence of H_2_DCFDA, expressed as percentages compared to controls after a 24 h treatment with CisPt 20 µM and/or the G. tub. extract (10 µg/mL); the G. par. extract (10 µg/mL) or the G. tub. and G. par. association extract (each at 5 µg/mL). Results are shown as mean ± SD of three independent experiments (**** *p* < 0.0001 compared to controls; #### *p* < 0.0001 compared to CisPt 20 µM). The signs + and − indicate the presence or the absence of the corresponding treatment respectively.

Scavenging activities at the working concentration (10 µg/mL) as well as IC_50_ have been determined using the DPPH assay. The results obtained are shown in Figure 3a. IC_50_ scavenging activities for G. tub., G. par., and their association were 124 ± 4 µg/mL, 13.8 ± 0.8 µg/mL, and 21.6 ± 0.8 µg/mL, respectively. Residual DPPH at the working concentration were 95.7%, 59.9%, and 72.9%, respectively.

The effect on the ROS’s increase due to CisPt incubation with or without the extracts is shown in Figure 3b. ROS production was not significantly affected by the extracts alone, whereas it was significantly increased by CisPt, up to 124 ± 8%. This ROS overproduction was significantly prevented by the G. par. methanolic extract, as reflected by a significant reduction to 112.4 ± 8%. The G. tub. and the association did not significantly modify the ROS’s production compared to the effect obtained with CisPt alone.

### 3.4. Anti-Inflammatory Measurement: Impact on the NFkB, KIM-1, IL-6 Markers

**Figure 4:**
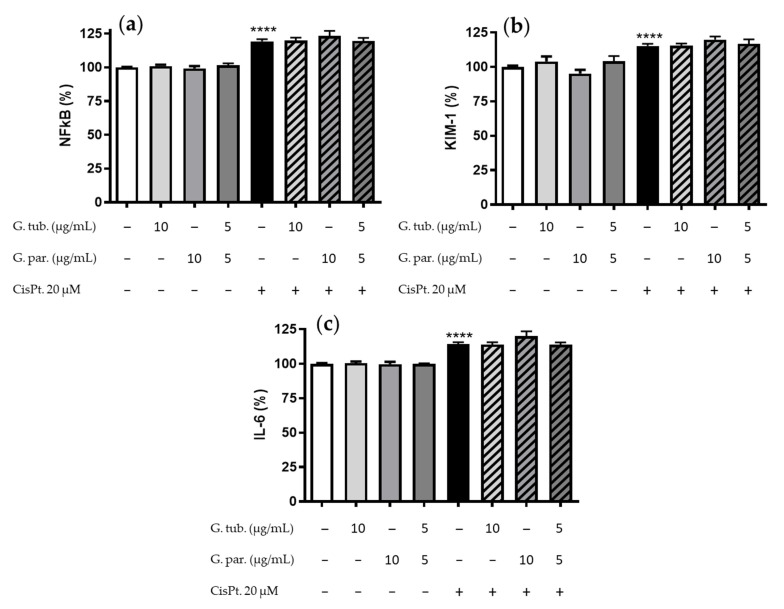
Anti-inflammatory measurements of immunostained cells after a 24 h treatment with CisPt 20 µM and/or the G. tub. extract (10 µg/mL); the G. par. extract (10 µg/mL) or the G. tub. and G. par. association extract (each at 5 µg/mL). Three markers were analyzed and their normalized fluorescences compared to controls are shown as follows: (**a**) intracytoplasmic NFκB proportion, (**b**) membranous KIM-1 proportion, and (**c**) intracytoplasmic IL-6 proportion. Results are shown as mean ± SD of three independent experiments (**** *p* < 0.0001 compared to controls). The signs + and − indicate the presence or the absence of the corresponding treatment, respectively.

The anti-inflammatory potential effect of the two mushroom extracts and their association was tested using three markers, namely NFκB, KIM-1 and IL-6. The results obtained are shown in Figure 4. None of the extracts, used alone, significantly modified the expression of these three markers. By contrast, CisPt exposure led to an increased expression of NFκB, KIM-1, and IL-6 in proportions to 119 ± 7%, 115 ± 6%, and 114 ± 5% compared to the controls, respectively (Figure 4a–c). In the test conditions, neither G. tub., G. par. extracts, nor their association could significantly prevent the increase in NFκB, IL-6, and KIM-1 proportions.

### 3.5. Intracytoplasmic Calcium Estimation

**Figure 5:**
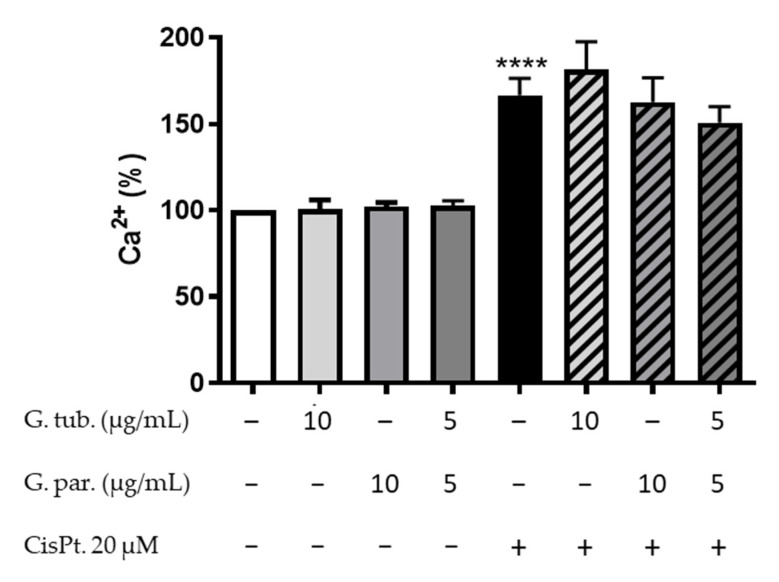
Intracytoplasmic calcium proportion of Fluo-3 AM dyed cells after a 48 h treatment with CisPt 20 µM and/or the G. tub. extract (10 µg/mL), the G. par. extract (10 µg/mL) or the G. tub., and G. par. association extract (each at 5 µg/mL). Cells were analyzed by flux cytometry, and results are shown as mean of normalized fluorescence compared to controls ± SD of three independent experiments (**** *p* < 0.0001 compared to the control). The signs + and − indicate the presence or the absence of the corresponding treatment, respectively.

The estimation of intracytoplasmic calcium after 48 h was determined in order to evaluate the dysregulation in late cell homeostasis. The results obtained are shown in Figure 5. The mushroom extracts did not significantly affect the intracytoplasmic calcium compared to the controls. CisPt, on the other hand, increased the proportion of intracytoplasmic calcium up to 166 ± 22%. A pre-treatment with the mushroom extracts could not prevent the CisPt-induced increase.

### 3.6. Intracytoplasmic β-Catenin Determination

**Figure 6:**
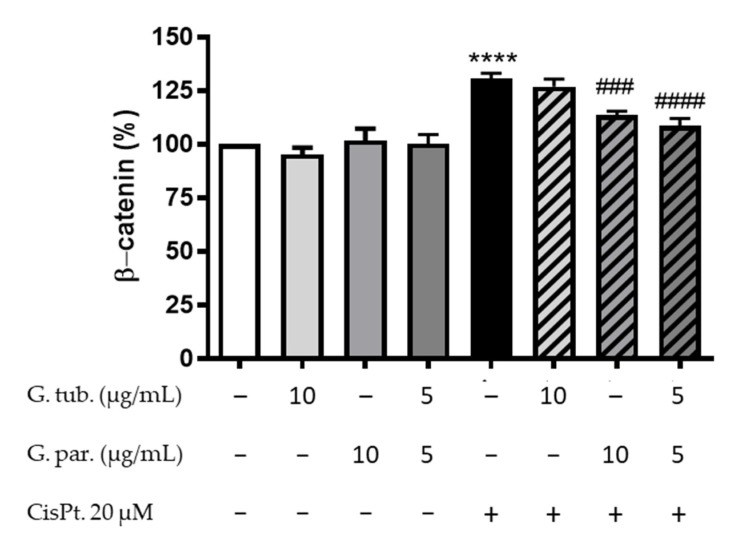
Intracytoplasmic β-catenin immunostained cells after a 24 h treatment with CisPt 20 µM and/or the G. tub. extract (10 µg/mL), the G. par. extract (10 µg/mL) or the G. tub., and G. par. association extract (each at 5 µg/mL). Cells were analyzed by flux cytometry and results are shown as mean of normalized fluorescence compared to controls ± SD of three independent experiments (**** *p* < 0.0001 compared to controls; #### *p* < 0.0001 and ### *p* < 0.001 compared to CisPt. 20 µM). The signs + and − indicate the presence or the absence of the corresponding treatment, respectively.

The intracytoplasmic proportion of β-catenin measured in all conditions is shown in Figure 6. The mushroom extracts did not significantly affect its proportion. However, CisPt exposure increased it up to 131 ± 11% compared to the controls. This increase was not prevented by pre-treating cells with G. tub. (127 ± 9%), but it was significantly prevented by G. par. (113 ± 4%) and by the association of both extracts (109 ± 8%).

### 3.7. Chemical Content

#### 3.7.1. Phytochemical Screening

The phytochemical screening results are shown in Table 1. All methanolic extracts showed the presence of terpenes. G. tub. 1, G. app. 1, G. tub. 2, G. par., and G. app. 2 showed the presence of simple reducing carbohydrates. Moreover, saponins were detected in G. par. and G. app. 1 methanolic extracts. Finally, the presence of tannins was suspected in G. app. 1, G. par., and G. app. 2 (change in color of the solution but no precipitate was observed).

#### 3.7.2. Total Phenolic and Triterpenoid Contents

Both TPC and TTC are shown in Table 2. Results have shown that G. app. 1 and G. par. have a higher TPC, with 121 ± 14 and 201 ± 12 mg of Gallic Acid Equivalent (GAE) per gram of extract, respectively. Both G. tub. and G. par., have a TTC over 200 mg Oleanolic Acid Equivalent (OAE) per gram of extract.

## 4. Discussion

Cisplatin is a currently and frequently used cancer treatment and is well known in its induction of tubulotoxicity [5]. As previously described by others, the present study confirms that CisPt has various deleterious effects on proximal tubular epithelial cells [3,5,28]. It activates the apoptosis pathway, dysregulates the late calcium homeostasis, increases the oxidative stress as well as pro-inflammatory and stress markers, and finally increases β-catenin translocation. Previous studies have shown the impact on various biomarkers in vitro in CisPt-induced nephrotoxicity on HK-2 cells. The results demonstrated that the apoptosis was induced, including a decrease in the Bcl-2 family and an increase on the activated caspase-3. Finally, the protein levels of KIM-1, calbindin, and TIMP-1 were also increased [28].

Numerous natural products have been tested for their potential positive impact on human health and among these, medicinal mushrooms are increasingly investigated. The *Ganoderma* genus and more specifically the *Ganoderma lucidum*, used in traditional Chinese medicine, have been studied in the case of CisPt nephrotoxicity in vivo as well as on cultured mice tubular epithelial cells. Results have introduced an interest in small molecules extracted from *G. lucidum*, as well as its methanolic and chloroformic extracts. The *G. lucidum* methanolic extract was used at 500 mg/kg, while the chloroformic extract was used at 100 mg/kg. In all studies, the *G. lucidum* extract has been given prior to CisPt injection. Interestingly, this extract exhibited antioxidant and anti-inflammatory activities and reduced apoptosis [13,15,29,30,31]. Finally, complex Chinese medicinal herb mixes (CMHs), containing *G. lucidum*, have been studied in CisPt-treated patients for non-small-cell lung cancer, and an improved median survival rate was reported. The authors related this result to a protective effect on the bone marrow hematopoietic system. Renal function parameters (serum levels of urea and creatinine) were not improved. The CMH was used as a decoction and results were observed [32].

Starting from these quite limited results, the present study aimed to increase the knowledge with respect to this genus by examining various *Ganoderma* species in vitro. Working concentrations were chosen based on a non-toxic concentration of the methanolic extracts, which was determined to be 10 µg/mL and 5 + 5 µg/mL when an association was considered. CisPt concentrations were chosen to reach about 85% of cell survival. The cell viability assay results confirmed the interest on *Ganoderma tuberculosum*, *Ganoderma parvigibbosum*, and their association as they prevented decreases in cell viability compared to cells exposed to CisPt alone. Following this, the investigations focused on the mechanisms of tubulotoxicity prevention by using the corresponding extracts.

**Apoptosis prevention.**

CisPt is a known pro-apoptotic xenobiotic drug. It induces apoptosis at micromolar concentrations and necrosis at millimolar concentrations using different mechanisms [13,33]. Previous studies have shown the apoptosis induction of CisPt on HK-2 cells. Indeed, their results showed an increase of up to 14.2% of apoptotic cells, which are comparable to our results (14.9%) [28]. CisPt is known to induce DNA damages as alkylating agent and to induce cellular stress by increasing ROS production [4]. The results of the present study confirmed the significant CisPt-induced apoptosis of cultured HK-2 cells. They also showed that G. tub., G. par., and the extract association prevented this apoptosis induction, using the annexin V/PI assay to determine and quantify the total apoptosis.

This prevention was observed at different steps of the apoptosis induction: p53, a transcriptional protein, can be early activated by a stimulus, such as a DNA damage or cellular stress. It is followed by a cytochrome C release from the mitochondria. Both result in the activation of the caspase pathway [33,34] and cell death [35,36,37]. Finally, caspase-3, a protease involved at the end of the proapoptotic signaling pathway is activated, leading to an enhanced apoptosis. It is, therefore, interesting for new potential cancer treatments [38,39]. In our in vitro study, G. par., G. tub., and their association were able to mitigate CisPt-induced apoptosis, including the increase in caspase 3.

Our in vitro results confirm prior in vivo observations showing the crucial impact of CisPt in apoptosis regulation. Mahran et al. demonstrated that the caspase 3 increase was significantly reduced by *G. lucidum* (about 0.5 fold compared to the CisPt group) [13]. Other authors reported the potential interest of *G. lucidum* small molecules through molecular docking. They have shown their capacity to inhibit the activation of the caspase-1 [31]. Finally, an ethanolic extract of *G. lucidum* has also shown the prevention of apoptosis in cadmium intoxicated chicken. Data of this in vivo study have demonstrated its ability to prevent the increase in caspase-3, Bax, and Bcl-2, all markers of the pro-apoptotic pathway in chickens’ spleen [40].

**Antioxidant and anti-inflammatory activities.**

As the production of ROS induces apoptosis signal transduction and activates apoptotic proteins, such as caspase 3, the antioxidant property of the mushroom extracts was studied [41]. Our results have shown a partial scavenger activity of the *G. parvigibbosum* extract at working concentrations. These results can be linked to its total phenolic content as polyphenols are known to show antioxidant activity [26]. Comparable results were observed with the association of the two extracts while *G. tuberculosum* did not exhibit this activity. Interestingly and logically, the ROS overproduction due to CisPt was prevented by the G. par. extract, linkable to its scavenger activity. Indeed, a scavenger activity can prevent the harmful effect of ROS increase measured in intoxicated cells and, therefore, decrease the induced toxicity [42]. Previous studies have shown that *G. lucidum* extracts are antioxidants in vivo. They have shown the ability to prevent superoxide dismutase, catalase, and glutathione peroxidase decreases as well as to restore glutathione and malondialdehyde levels [29,30]. It was also shown that the H_2_O_2_ renal level was reduced by 1.8 fold in pre-treated animals prior to CisPt administration [13].

CisPt is also known to induce a pro-inflammatory response in intoxicated cells [5,6], including in HK-2 cells. To further examine this, three markers of the pro-inflammation cell response were studied: the intracytoplasmic increase in NFκB, IL-6, and the membranous overexpression of KIM-1. NFκB is a transcription factor that has a key role in immune and inflammatory responses [43,44]. IL-6 is an inflammatory cytokine that is overexpressed in response to stimuli, allowing an immune reaction of cells [45]. KIM-1 is a specific glycoprotein mainly expressed by proximal tubule cells, and it is upregulated when cells are exposed to a nephrotoxin, allowing a response to the injury [46,47]. As *G. parvigibbosum* prevented ROS overproduction, it could have decreased pro-inflammatory responses. In our hands, despite observing antioxidant activities, no anti-inflammatory response due to the pre-treatment of the cells with the mushroom’s extracts was detected. These results actually contrast with previous studies that report the capacity to prevent the CisPt-mediated inflammatory signal through NFκB [13,31], interleukin 1 [31], and the high-mobility group box-1 [13]. These studies were conducted in vitro with a purified small molecule from *G. lucidum* [31] or in vivo in mice receiving *G. lucidum* [13]. However, it must be noted that the results from Xue et al. are preliminary results, and further information on the results are needed.

**Effect on the late calcium homeostasis.**

The late calcium homeostasis dysregulation observed by the addition of CisPt can be an important cause of the ROS overproduction and can lead to cell damages [48,49]. It is known that CisPt treatments lead to mitochondrial and endoplasmic reticulum dysfunctions, including a decrease in calcium uptake; thus, an increase in intracytoplasmic calcium concentration [6]. Our study contributed to demonstrating that CisPt was able to interfere with the long-term homeostasis of calcium in vitro but the mushroom extracts were not able to prevent it.

**Effect on the β-Catenin translocation.**

β-Catenin is a protein that is known to play a role in cell adherence and proliferation, apoptosis, and pro-inflammatory-associated cancer through the Wnt/β-catenin signaling pathway. Inhibiting this pathway can mitigate renal fibrosis [50]. β-catenin is mostly located on the cytoplasmic side of the membrane before being translocated to the nucleus in the presence of a stimulus [51,52,53]. While these stimuli can be numerous, external stimuli, such as xenobiotics, and more specifically CisPt can lead to the delocalization of the β-catenin, hence increasing its intracytoplasmic concentration and resulting in higher fibrosis-occurring risks [54]. Interestingly, in our in vitro study, G. par. and the association of G. par. and G. tub. were able to prevent this internalization. The inhibition of β-catenin translocation by the mushrooms extracts may be due to their antioxidant potential since ROS can activate the Wnt/β-Catenin signaling pathway [55]. Their antiapoptotic potential could also favor cell adhesion, thereby preventing β-cat translocation following loss of E-cadherin. That type of protection may therefore lead to a lower risk of cell inadherence and fibrosis [51,52,53].

**Chemical content**

The *Ganoderma* genus is known to be rich in acid triterpenes and polysaccharides [56,57]. Triterpenes have been widely studied for various applications such as antidiabetic, antiviral and anticancer properties [13,16,17]. The species itself as well as the extracts tested lead to a better understanding of the observed activity. Indeed, the polysaccharides of *G. lucidum, G. sinense*, and *G. microsporum* exhibit an enhanced apoptosis in cancer cells in association with CisPt [58,59,60]. Our study has shown that the five species studied contain terpenes when performing the phytochemical screening. Most of the mushrooms also contain simple reducing carbohydrates. TTC determination indicates that a higher concentration of triterpenoids increases the protective effect on healthy HK-2 cells exposed to a nephrotoxicant. Interestingly, although CisPt is well-known to induce a pro-inflammatory and a pro-oxidant response [5], the total phenolic content, linkable to the antioxidant activity, does not allow a decrease in cell death. Indeed, while G. app. 1 has thrice the amount of polyphenols than G. tub. 1 and 2, it was unable to prevent CisPt-induced tubulotoxicity. The combination of a higher TTC and TPC, as for G. par., is more interesting. Indeed, it not only significantly decreased the total apoptosis and its signaling pathway, likewise observed for G. tub., but also decreased the internalization of β-catenin and prevented the ROS overproduction. These promising results highlight the importance of the triterpenoid content of the *Ganoderma*’s genus and their potential activity. Metabolomic and analytical studies are currently conducted in order to further identify the chemical composition of the extracts and to understand the link between the chemical compounds and the observed cell’s protective activities.

## 5. Conclusions

All together, these results confirm the promising nephroprotective effect of *Ganoderma parvigibbosum* and *Ganoderma tuberculosum* against in vitro CisPt tubulotoxicity. The results show the importance of continuing the study of new species. The prevention of this toxicity can be mainly explained by the capacity to downregulate the pro-apoptotic pathway. Both methanolic extracts can therefore be useful as adjuvant agents. In order to confirm this, more studies will be required to analyze the chemical composition and the cell selectivity of the protective properties observed. Tests on cancer cells should be performed.

## Figures and Tables

**Table 1:** Phytochemical screening results of methanolic extracts of the 7 studied specimens. The signs +, ± and − indicate the presence, the suspected presence or the absence of the corresponding secondary metabolite, respectively.

Secondary Metabolite	G. tub. 1	G. app. 1	G. tub. 2	G. par.	G. mar.	G. res.	G. app. 2
Simple carbohydrates	**+**	**++**	**+**	**++**	−	−	**++**
Terpenes	**+**	**+**	**+**	**+**	**+**	**+**	**+**
Saponins	−	**+**	−	**+**	−	−	−
Alkaloids	−	−	−	−	−	−	−
Flavonoids	−	−	−	−	−	−	−
Tannins	−	±	−	±	−	−	±

**Table 2:** Total phenolic and triterpenoid contents in the methanolic extracts of the 7 specimens studied.

	Total Phenolic Content ^1^	Total Triterpenoid Content ^2^
G. tub. 1	47 ± 4	212 ± 12
G. app. 1	121 ± 14	110 ± 13
G. tub. 2	40 ± 3	256 ± 13
G. par.	201 ± 12	261 ± 16
G. mar.	26 ± 2	138 ± 12
G. res.	30 ± 2	169 ± 14
G. app. 2	77 ± 7	157 ± 15

^1^ mg GAE/g extract. ^2^ mg OAE/g extract.

## Data Availability

Not applicable.
